# *Kiwifruit*’s Allergy in Children: What Do We Know?

**DOI:** 10.3390/nu15133030

**Published:** 2023-07-04

**Authors:** Ivana Bringheli, Giulia Brindisi, Rebecca Morelli, Lavinia Marchetti, Ludovica Cela, Alessandro Gravina, Francesca Pastore, Antonio Semeraro, Bianca Cinicola, Martina Capponi, Alessandra Gori, Elia Pignataro, Maria Grazia Piccioni, Anna Maria Zicari, Caterina Anania

**Affiliations:** Department of Maternal Infantile and Urological Science, Sapienza University of Rome, 00161 Rome, Italy; ivana.bringheli@uniroma1.it (I.B.); giulia.brindisi@uniroma1.it (G.B.); rebecca.morelli@uniroma1.it (R.M.); lavinia.marchetti@uniroma1.it (L.M.); ludovica.cela@uniroma1.it (L.C.); alessandro.gravina@uniroma1.it (A.G.); f.pastore@uniroma1.it (F.P.); antonio.semeraro@uniroma1.it (A.S.); bianca.cinicola@uniroma1.it (B.C.); m.capponi@uniroma1.it (M.C.); alessandra.gori85@gmail.com (A.G.); elia.pignataro@uniroma1.it (E.P.); mariagrazia.piccioni@uniroma1.it (M.G.P.); annamaria.zicari@uniroma1.it (A.M.Z.)

**Keywords:** *kiwifruit*, children, allergy, cross-reactivity, Act d 1

## Abstract

*Kiwifruit* allergy is an emerging pathological condition in both general and pediatric populations with a wide range of symptoms linked to variable molecular patterns, justifying systemic and cross-reactions with other allergens (i.e., latex, pollen, and fruit). Skin prick test (SPT), specific serum IgE (Act d 1, Act d 2, Act d 5, Act d 8, and Act d 10) directed against five out of thirteen molecular allergens described in the literature, and oral test challenge with *kiwifruit* are available for defining diagnosis. The management is similar to that of other food allergies, mostly based on an elimination diet. Although kiwi allergy has been on the rise in recent years, few studies have evaluated the clinical characteristics and methods of investigating this form of allergy. Data collected so far show severe allergic reaction to be more frequent in children compared to adults. Therefore, the aim of this review is to collect the reported clinical features and the available association with specific molecular patterns of recognition to better understand how to manage these patients and improve daily clinical practice.

## 1. Introduction

*Kiwifruit* (*Actinidia* spp.) belongs to the family *Actinidiaceae* and genus *Actinidia*. At the beginning of the nineteenth century, the plant was detected for the first time in China, growing wild along the Yangtze River valley. In 1904, the seeds were brought to New Zealand and its fruits were referred to as the “Chinese gooseberry”. When New Zealand began the exportation, around the 1960s, the name was changed to kiwi as the kiwi bird is the New Zealand national emblem [1]. Nowadays, the main producing countries are China, Italy, New Zealand, Chile, and Greece, covering 87% of the worldwide need. According to the United States Department of Agriculture (USDA) Nutrient database, *kiwifruit* is rich in fiber, carbohydrates, vitamin C and E, minerals, omega-3 fatty acids, and antioxidants [2]. The concentration of Actinidin varies with the maturity of the fruit, from low to high concentration during postharvest ripening. There are more than 60 species of kiwi, and of which, the main ones marketed to date are derived from 4 main species: *A. deliciosa* (including the most common “Hayward” kiwis), which are large and have green pulp; *A. chinensis*, which are large and whose pulp can be green, yellow, or red; *A. arguta*, which are small, hairless, and soft; and finally, the *A. eriantha*, which are long, cylindrical, and hairy. (Table 1) *Actinidia deliciosa* is the most cultivated and widespread species on a global scale [3].

## 2. Methods

Search engines, such as PubMed and Scopus server, were used as the main sources of information in writing this review. The following keywords were applied: “*kiwifruit*, allergy, children, *Actinidia*, cross-reactivity”. Studies were evaluated from 1981 to the present, including case reports, observational studies, retrospective studies, and previous reviews on the same topic.

## 3. Epidemiology

Acute allergy to *kiwifruit* was first described relatively late in 1981, by Fine [1], and nowadays it has become a major elicitor of plant food allergies. Nevertheless, the global statistics regarding the distribution of *kiwifruit* allergy are limited. Most research works have been conducted on the European population, although data regarding children are still lacking. In France, Rance et al., among 182 children (from 2 to 14 years of age) with a consistent history of food allergy, found that 9% were sensitized to *kiwifruit* [4]. The literature reports that *kiwifruit* allergies are often cross-reactive with others such as pollen, rye, hazelnut, chestnut, banana, and avocado. The large amount of birch trees located in Europe may elicit allergies through the cross-reactivity between birch pollen and the homologous structures found in *kiwifruit* protein; this may explain the larger amount of research works conducted mainly on adult European patients [5,6]. In their study, Le et al. analyzed *kiwifruit* allergen sensitization patterns and clinical manifestations across Europe (12 countries) in consideration of four different climatic conditions, revealing marked differences. Patients from Iceland (the only representative of northern Europe) mainly experienced severe symptoms (respiratory and cardiovascular), while most patients from central/western and southern Europe predominantly showed oral allergic syndrome (SOA). Furthermore, *kiwifruit* allergy associated with birch pollen allergy was most depicted in central/western Europe, while in southern Europe, the association was the highest with grass pollen allergens. Mono-allergy to *kiwifruit* was most frequently found in Iceland and southern Europe. The clinical differences between these groups have been linked to specific molecular patterns which will be further discussed in this review [7]. As for children, they are more likely to react on the first known exposure and more frequently than adults who develop systemic manifestations [8]. Additionally, although *kiwifruit* allergy is frequently associated with grass and birch pollen allergies, children are often mono-sensitized to *kiwifruit*, suggesting a role of primary digestive tract sensitization and a different pattern of IgE recognition of *kiwifruit* proteins than in adults [9]. 

## 4. Molecular Allergens

Until now, thirteen allergens have been identified in *kiwifruit*; however, not all are accessible via in vitro diagnosis in clinical practice [10]; Table 2 below will resume their main characteristics. By far, Actinidin, also named Act d 1, represents the major allergen, since it stands for about 50% of the total soluble protein content [11]. It is frequently found in mono-sensitized patients not allergic to pollen, and is associated with the most severe symptoms. Its effect on intestinal epithelial cells is being studied. It is known that Act d 1 acts as a cysteine protease and causes a breach in the epithelial barrier, thus playing an important role in the sensitization process to *kiwifruit* [12]. Act d 2 is a thaumatin-like protein, and a similarity between Act d 2 and Alt a 1 has been observed both in vivo and in vitro [13]. Alt a 1 is the major allergen of Alternaria, a mold also known for the risk of fruit contamination. In fact, by penetrating the pulp, the protein Alt a 1 can establish electrostatic interactions (hydrophilic, hydrophobic, or both) with Act d 2. These interactions may cause interference with the detention capacity of dedicated diagnostic tests to detect specific IgE directed toward the native Act d 2 molecule. This has made its sensitizing prevalence unclear, given that mono-sensitization to Act d 2 is extremely rare. The clinical relevance of this association was confirmed by the high frequency of co-sensitizations observed between Act d 2 and Alt a 1 via a multimolecular test of ISAC microarrays (immuno solid-phase allergen chip): up to 85% of patients sensitized to Act d 2 were also sensitized to Alt at 1. In contrast, only 39% of patients sensitized to Alt a 1 were co-sensitized to Act d 2. Similarly, Alt a 1 could be associated with thaumatin-like PR5 proteins found in other fruits, such as cherry (Pru av 2), apple (Mal d 2), and banana (Mus a 4) [14]. Act d 3 is a 45 kDa glycoallergen, called chitinase, possibly implicated in latex fruit syndrome [15]. Act d 4 has a role in the inhibition of cysteine proteinases [16], but its clinical relevance is still unclear. Act d 5, also called Kiwellin, is a cystein-rich protein, and studies in vivo and in vitro show that a proteolytic process could split it into two additional proteins (KiTH and kissper) thanks to *kiwifruit* actinidin [17]. Act d 6 is a pectin methyl esterase inhibitor that possibly takes part in the regulation of the ripening of the fruit. Act d 7 is a pectin methylesterase allergen that only a small number of allergic patients are affected by [18]. Act d 8, in its composition, is very similar to Bet v 1, birch pollen’s major allergen, and it is in fact considered a pathogenesis-related protein class 10 (PR-10); this partially explains the cross-reaction, which will be discussed later on [19]. Act d 9 is a profilin which acts as a panallergen, and it can be intercepted in patients allergic to grass as well. Act d 10 is a nonspecific lipid transfer protein (LTP) and it is considered to be a minor allergen, like Act d 9 and Act d 11 [18]. Act d 11, since it is a major latex protein/ripening-related protein, usually cross-reacts with proteins of the PR-10 family [20]. Recent studies have found two new proteins in the composition of *kiwifruit*’s seeds: Act d 12 and Act d 13, a major and a minor allergen which share common epitopes from peanut and tree nuts, respectively, suggesting cross-reactivity with them [21,22].

## 5. Pathophysiology

Gut-associated lymphoid tissue is composed of innate immunity cell populations which normally react to dietary proteins with the induction of oral tolerance, which is an active inhibition of immune responses toward ingested food [23]. 

The pathophysiology of cross-reactions, instead, could be explained by molecular mimicry. Different allergens may share a similar stereometric conformation, which might as well trigger the mast cells by binding the IgE at the surface. Therefore, clinically significant reactions, which account for mild, moderate, or severe responses, either follow direct sensitization to the specific allergen or cross-reactions with other allergens of similar structures. The severity of symptoms is, in part, dependent upon the route of sensitization with the highest risk of severe anaphylactic reactions in those patients primarily sensitized to the allergen [23].

## 6. Clinical Manifestations

As with every food-related allergy, clinical manifestations vary from mild–moderate to severe reactions. Table 3 highlights the main clinical reports of *kiwifruit* allergy conducted from 1981 to date. The reports concerning the pediatric population are limited. Most of the information gathered in the literature has been primarily found in studies designed to explore *kiwifruit* cross-reactivity with other allergens in adults. Usually, the onset of symptoms occurs 2 h after kiwi exposure (ingestion or direct contact), since an IgE-mediated pattern is involved. A complete resolution of the reaction occurs within several hours. The large spectrum of clinical manifestations includes cutaneous, gastrointestinal, respiratory, cardiovascular, and neurological signs and symptoms in an isolated or concomitant manner with the same or different timing. Mild–moderate reactions might be distinguished by itching and tingling of the lips, oral mucosa, and/or itching and tingling of the tongue (oral allergy syndrome). Instead, severe reactions include urticaria or angioedema, contact urticaria, laryngeal swelling, immediate vomiting, rhinitis, cough, wheezing, bronchospasm, hypotension, loss of consciousness, and even food-dependent exercise-induced anaphylaxis [8]. As previously noted, children are more likely to develop systemic reactions involving more organs at once. For instance, Shimizu and Morikawa reported a case of a 12-year-old boy with atopic dermatitis and allergic rhinitis who developed anaphylaxis after 15 min from the second ingestion of kiwi in his life. The kiwi-specific IgE level and the skin prick test were positive [24]. Also, Rance et al. reported a case of a 3-year-old boy and an 8-year-old girl rapidly developing hypotensive shock after handling *kiwifruit* [25]. A recent Italian study including 25 patients confirmed that the most frequent symptoms are angioedema and urticaria followed by AOS, gastrointestinal symptoms, rhinoconjunctivitis, cough, and dyspnea, while anaphylaxis was described in six patients. All of the patients tested positive for the allergen extracted via the skin tests and for specific IgE [26]. The severity of reactions is useful for suspecting the precise molecular pattern of sensitization involved. Being able to distinguish, on a molecular basis, between primary or cross-reactive reactions nowadays represents a great resource for proper management.

## 7. Cross-Reactions

*Kiwifruit* cross-reactions are well described in the adult population, especially for birch and grass pollen, avocado, banana, latex, and hazelnut [32]. Indeed, pre-sensitization to birch pollen or grass predispose an individual to kiwi allergy, mostly via sensitization to Act d 8, PR-10 of kiwi, and homolog of Bet v1, which is the major allergen of birch [30]. Aleman et al. conducted a double-blind, placebo-controlled food challenge (DBPCFC) study to identify the patterns of allergen recognition in kiwi-sensitized patients from a birch-free area. No definite allergen recognition pattern was associated with the type of allergic reaction to kiwi. One in five patients with kiwi allergy was not allergic to pollen, and this patient had the highest risk of systemic reaction to kiwi. It seems that the subjects with associated pollen allergy are likely to have oral allergy syndrome, while it appears that subjects with an allergy to *kiwifruit* in the absence of pollinosis are more likely to have systemic symptoms and anaphylactic shock [33]. Another interesting study conducted in Japan on pediatric patients (median age at onset of symptoms: 6 years) by Tomoyuki et al. identified a negative specific IgE response to Act d 8 (PR-10 equivalent) as a risk factor for a severe allergic reaction in children sensitized to kiwi. In other words, the severity of *kiwifruit* allergy is related to the sensitization of PR-10: several children with *kiwifruit* allergy without sensitization of PR-10 will present severe allergic symptoms [22]. Concerning latex, it is well known that around 30–50% of patients present concomitant hypersensitivity to certain foods of plant origin, including kiwi. This condition is called “latex-fruit syndrome” and can be explained by the common epitopes that *kiwifruit* share with latex, avocado, and banana in particular [29]. There are no studies of an adequate size to further investigate the differences between subjects with mono-allergy, pollinosis, or latex-associated symptoms. We do not know whether children respond differently from adults, and the natural history of the allergy is mostly unknown.

## 8. Diagnosis

**Figure 1:**
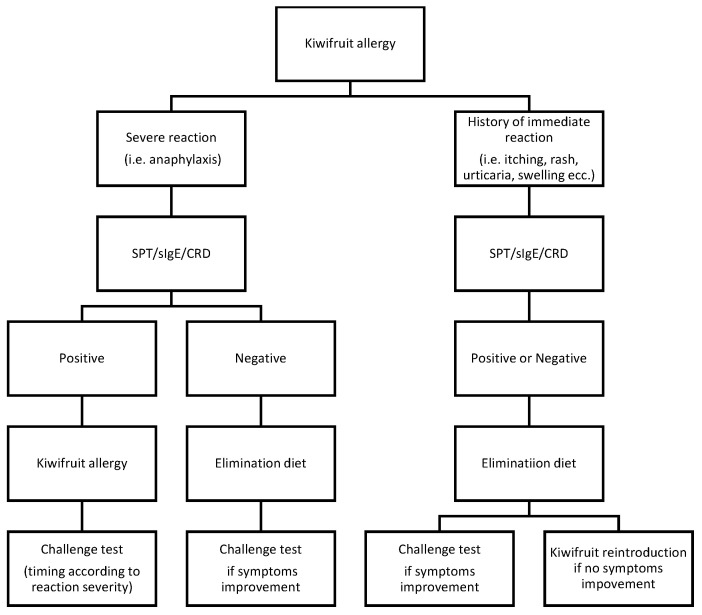
Diagnostic flowchart. Patient personal histories of severe reactions need to be tested via SPT, RAST, and CRD. Anaphylaxis is indicated by “severe reaction”. In this case, if the laboratory test result is positive, a food allergy is claimed; the timing for the challenge test is decided after considering the clinical response and the outcome of the CRD test. If the serological exams are negative, a focused elimination diet should follow. Based on the diet change response, the challenge test becomes worthy in revealing the diagnosis. The diagnostic process is similar in patients with immediate nonsevere reactions after *kiwifruit* ingestion, such as rash, urticaria, dyspnea, intense coughing, swelling of tongue, lips, and extremities, and itching. Apart from the results of the diagnostic tools, an elimination diet should be implemented. If relief from symptoms is achieved, the challenge test should be performed; on the contrary, it is possible to reintroduce the food, especially with negative serum diagnostic test results (including the CRD) [40]. In this case, there is no evidence to claim the specific *kiwifruit* allergy.

Patient clinical history and examination are the first steps toward a diagnosis of *kiwifruit* allergy [34]. Other clinical diagnostic tools include skin prick test (SPT), enzyme-linked immunosorbent assay (ELISA), component-resolved diagnosis (CRD), and double-blind placebo-controlled food challenge (DBPCFC) [35]. DBPCFC is considered to be the gold standard test to detect a food allergy because it ensures an objective assessment of outcomes without operator-specific preconceptions or biases [36]. However, it is logistically demanding, and anaphylactic reactions may occur. Therefore, if the anamnesis is suggestive, an SPT is performed as a more accessible diagnostic tool to identify IgE-specific sensitization (95%) [37]. D’Amelio et al. [38] found low sensitivity when an SPT was applied using commercial *kiwifruit* extracts (52.8–66.7%), while a prick by prick test with fresh *kiwifruit* yielded the highest sensitivity (81.8%); hence, it has been shown that it preserves kiwi allergenic proteins, ensuring good diagnostic capacity [17]. ELISA and immunoCAP (a commercial ELISA) are two similar blood test methods based on the detection of specific serum IgE. Reports to date are contradictory about the role of measuring specific IgE to confirm *kiwifruit* allergy. Lucas et al. [8] found the test to have good specificity (83%) and poor sensitivity (60%), with the latter being most probably related to the lability of allergens, as it had already been identified in the skin test solution. Furthermore, the study revealed that the level of specific IgE was not correlated with the reported severity of symptoms or age. It is noteworthy that serum IgE toward *kiwifruit* extract (ELISA/ImmunoCAP) corresponds to the detection rate of CRD in recognizing Act d 1, the major *kiwifruit* allergen [37]. Bublin M. et al., by applying CRD, managed to classify adult patients in different reactor groups: [16] actinidin (Act d 1) may serve as a marker for isolated *kiwifruit* allergy, while Act d 8 and Act d 9 might be indicative of typical cross-reactivity patterns. As previously described, the clinical association of pollinosis with an allergy to fresh fruit, including kiwi, is well recognized indeed [39]. Many allergens in *kiwifruit* are readily digested by simulated gastric fluid; this may explain why most allergic reactions are restricted to the oral cavity (OAS) in adults with a pollen allergy. The aforementioned study on a cohort of 25 Italian children also specified that the allergens (Act d 8, Bet v1) linked to pollen–fruit syndrome could also be associated with sensitization to Act d 1, the major stable antigen, thus explaining the risk of severe systemic reactions [26]. Moreover, the potential gastrointestinal route of sensitization could expose different allergens or combinations of allergens from *kiwifruit* that may elicit more severe clinical manifestations in children [9]. Therefore, the introduction of CRD represents a useful tool which needs to be further investigated in the diagnostic process toward a definition of *kiwifruit* allergies. The following diagnostic flowchart (Figure 1) underlines the role that CRD should have in managing *kiwifruit* allergy. As children seem to be more affected by systemic reactions, analyzing the molecular status related to the allergen of each patient could predict the personal risk for life-threatening events or mild reactions, thus allowing for better management of the disease. 

## 9. Prevention and Therapy

Unlike allergies to pollen, currently there are no immunotherapy options to cure food allergies. Some strategies to induce changes in allergen confirmation and their IgE binding activity are under investigation. Several studies have shown that different thermal processing methods (such as sterilization, microwave heating, boiling, and steam cooking) or high-intensity ultrasound (20 to 100 kHz) treatments, as well as chemical processing (application of enzymes or ethylene) could reduce food allergenicity [41]. However, it is challenging to find a balance between a reduction in food allergenicity and the maintenance or improvement of food quality by applying processing technologies. 

Therefore, *kiwifruit* allergy still requires, as with other food allergies, a complete abstinence from the ingestion of the fruit. For mild, local reactions, first-line treatment is represented by antihistamines which prevent the action of activated mast cells. When it happens that two organ systems are involved in the allergic reaction, epinephrine is required. This molecule is also known as adrenaline, is available as a self-injection medication, and has been shown to be highly effective and well managed by either the same patient or their own family. Adrenaline has an effect both on the alpha and beta adrenergic receptors of the sympathetic system. Thus, on the one hand, there is a positive regulation of glycogenolysis, glucagon, and adrenocorticotropic hormone secretion, as well as lipolysis enhancement. The sum of these actions improves blood pressure, airflow, and respiratory function, thus solving the anaphylactic condition. The idea of a specific immunotherapy to induce food tolerance would be a great opportunity to improve the quality of life of these patients. By gradually increasing the exposition to offending allergens, patients’ levels of tolerance improve until no more immune responses are elicited. Unfortunately, this approach is still time-consuming and carries significant risks of anaphylaxis during the desensitization of food allergies. Moreover, although symptoms related to the inhalation of different pollens during the allergy season are reduced via the desensitization approach of immunotherapy, a concomitant significant improvement in OAS has not been proven for most patients. In addition, long-term efficacy is limited because benefits usually diminish when treatment is discontinued [40]. An interesting therapeutic option is initially emerging in trials evaluating the association between oral immunotherapy and anti-IgE monoclonal antibodies to induce food desensitization [42]. Only a few studies on this approach are currently available but these are not sufficient to assess the efficacy and safety of such a combination treatment. Moreover, most of the trials have been designed for severe cow’s milk protein allergy, since it represents the most common food allergy in young children, for which a tendency toward natural desensitization with growth is well known [43].

## 10. Conclusions

*Kiwifruit* allergy is emerging as a potential life-threatening condition, especially in the pediatric population which is more prone to primary sensitization to the fruit. The search for specific molecular antigens and their characterization has been a valid support in understanding the personal risk for severe manifestations after ingestion. More studies are needed to understand the age-based difference in sensitization to *kiwifruit* allergens. So far, the focused elimination diet remains the only available strategy.

## Figures and Tables

**Table 1:** Description of the main *kiwifruit* species.

Species	Shape	Density of Hairs	Fruit Flesh
*A. deliciosa*	Large, ovoid	Dense	Green
*A. chinensis*	Large, elliptical	Sparse or absent	Yellow, green, or red
*A. arguta*	Small, round	Absent	Green
*A. eriantha*	Long, cylindrical	Abundant, long, villose, white	Dark green

*A.* = *Actinidia*.

**Table 2:** *Kiwifruit* allergens and their main characteristics.

Allergen	Biochemical Name	Molecular Weight	Clinical Significance
Act d 1	Cysteine protease (actinidin)	30 kDa	Major allergen related to systemic reactions
Act d 2	Thaumatin-like protein	24 kDa	Major allergen
Act d 3		40 kDa	Minor allergen
Act d 4	Phytocystatin	11 kDa	Minor allergen
Act d 5	Kiwellin	28 kDa	Minor allergen
Act d 6	Pectin methylesterase inhibitor	18 kDa	Minor allergen
Act d 7	Pectin methylesterase	50 kDa	Minor allergen
Act d 8	Pathogenesis-related protein, PR-10, Bet v 1 family member	17 kDa	Major allergen, negative predictive risk factor for systemic reactions
Act d 9	Profilin	14 kDa	Minor allergen
Act d 10	nsLTP1	10 kDa	Minor allergen associated with mild symptoms, mainly in southern Europe
Act d 11	Major latex protein/ripening-related protein (MLP/RRP), Bet v 1 family member	17 kDa	Major allergen
Act d 12	Cupin, 11S globulin, *kiwifruit* seed storage protein	50,207.304 Da (mass spectrometry), six peptide sequences (C0HJF9, to be released upon publication)	Major allergen
Act d 13	2S albumin, *kiwifruit* seed storage protein	11,359.6 Da (mass spectrometry), four peptide sequences (C0HJG, to be released upon publication)	Minor allergen

**Table 3:** Main studies reporting clinical features related to *kiwifruit* adverse reactions in both pediatric and adult populations.

Author, Year [Reference]	Country	Study Design	Population and Sample Size	Comment
Fine et al., 1981 [1]	United States of America	Case report	A 53 y woman	First-reported allergic reaction to *kiwifruit*.
Rance et al., 1992 [25]	France	Case report	A 3 y boy and 8 y girl	New emerging allergies (kiwi) in children with systemic manifestations.
Shimizu et al., 1995 [24]	Japan	Case report	A 12 y boy	A case of anaphylaxis after handling and ingestion of *kiwifruit*.
Pastorello et al., 1996 [6]	Italy	Observational study	30 pts (16–69 y) with OAS to kiwi	Identification and characterization of 11 kiwi allergens.
Möller et al., 1997 [27]	Germany	Observational study	29 pts (23–57 y)	Cross-reactivity between allergens found both in *kiwifruit* and birch pollen.
Voitenko et al., 1997 [28]	Denmark	Observational study	9 pts (20–54 y)	Production of a kiwi extract and characterization of cross-reactivity with birch pollen in vitro.
Möller et al., 1998 [29]	Germany	Observational study	12 pts (20–58 y)	Cross-reactivity among allergens in latex, avocado, banana, and kiwi.
Fahlbusch et al., 1998 [30]	Germany	Observational study	9 pts	Characterization of IgE-binding *kiwifruit*’s allergen epitopes.
Gavrovic-Jankulović et al., 2002 [31]	Yugoslavia	Observational study	7 pts	Isolation and biochemical characterization of Act d 2.
Lucas et al., 2004 [8]	United Kingdom	Observational study	273 pts (4 months–71 y)	Clinical differences in children and adults.
Palacin et al., 2008 [15]	Spain		92 pts (6–55 y)	High IgE level against Act d 1 and Act d 3 is associated with severe symptomatology.
Moreno Álvarez et al., 2015 [9]	Spain	Observational study	24 pts (3–12 y)	Characterization of main allergens and patterns of recognition in children.
Visentini et al., 2018 [26]	Italy	Retrospective study	25 pts (1–14 y)	Primary sensitization to *kiwifruit* is the most frequent in childhood.

y = year old; pts = patients.

## Data Availability

Not applicable.
